# Phosphorus deficiency changes carbon isotope fractionation and triggers exudate reacquisition in tomato plants

**DOI:** 10.1038/s41598-020-72904-9

**Published:** 2020-09-29

**Authors:** Raphael Tiziani, Youry Pii, Silvia Celletti, Stefano Cesco, Tanja Mimmo

**Affiliations:** Faculty of Science and Technology, Free University of Bolzano, 39100 Bolzano, Italy

**Keywords:** Plant sciences, Environmental sciences

## Abstract

Plant roots are able to exude vast amounts of metabolites into the rhizosphere in response to phosphorus (P) deficiency. Causing noteworthy costs in terms of energy and carbon (C) for the plants. Therefore, it is suggested that exudates reacquisition by roots could represent an energy saving strategy of plants. This study aimed at investigating the effect of P deficiency on the ability of hydroponically grown tomato plants to re-acquire specific compounds generally present in root exudates by using ^13^C-labelled molecules. Results showed that P deficient tomato plants were able to take up citrate (+ 37%) and malate (+ 37%), particularly when compared to controls. While glycine (+ 42%) and fructose (+ 49%) uptake was enhanced in P shortage, glucose acquisition was not affected by the nutritional status. Unexpectedly, results also showed that P deficiency leads to a ^13^C enrichment in both tomato roots and shoots over time (shoots—+ 2.66‰, roots—+ 2.64‰, compared to control plants), probably due to stomata closure triggered by P deficiency. These findings highlight that tomato plants are able to take up a wide range of metabolites belonging to root exudates, thus maximizing C trade off. This trait is particularly evident when plants grew in P deficiency.

## Introduction

Phosphorus (P) is an essential macronutrient for all living organisms. Together with nitrogen (N), they are the two most important limiting factors for agricultural production^1^. Phosphorus is a key element of fundamental biomolecules such as ATP, nucleic acids, phospholipids and proteins. Already a latent P shortage can have a dramatic impact on plant growth and development^2^. In fact, the unavailability of this nutrient in the growing medium generally induces considerable morphological and physiological modifications in the plant^3^. A typical example of a morphological alteration is the increased root-to-shoot ratio as the result of a limited biomass production at shoot level^4^. In fact, root growth is usually only slightly restrained or, in some cases, even enhanced. Examples include species belonging to the Proteaceae family that can form the so-called proteoid roots, e.g. *Lupinus albus* L.^3^. However, this is not the only case, as there are other plant species that, when exposed to nutritional P disorder, prefer the growth of lateral roots and secondary branches instead of primary ones^5^. This enhanced root development is interpreted as an attempt of the plant to increase the soil volume explorable by roots for a better exploitation of the P enriched micro-domains and for a significant increase of P acquisition^3^. Concerning instead the physiological effects, P deficiency has a clear and relevant impact on photosynthesis^3,6,7^. The limitations of this anabolic processes have been reported in several plant species and crops, such as *Eucalyptus globulus*^8^, rice^9^, wheat^10^ or maize^11^. Two factors are held mainly responsible for this: (i) the role of P in the anatomy and functionality of stomata and (ii) the content and activity of ribulose-1,5-bisphosphate carboxylase/oxygenase protein (Rubisco)^12^. With respect to the first one, it is well known that P deficiency prevents healthy stomata functions, thus leading to a decrease of stomatal conductance^13^. Concerning instead the Rubisco protein, P deficiency impacts on both the activity of this enzyme and the transcription levels of the genes codifying for the protein. It is interesting to note that also the genes codifying for other photosynthetic enzymes involved in the metabolic pathway of the dark phase are down regulated in P shortage. Consequently, the whole photosynthetic process is negatively modulated and, from a general point of view, the entire C metabolism impaired with a significant impact on the whole development of the plant^6,14^.

Plant roots influence their surrounding soil volume through the release of a plethora of different high and low molecular weight compounds deriving from primary and secondary C metabolism named root exudates^15^. Root exudates are exuded actively through primary or secondary transport with ATP consumption or passively through diffusion, ionic channels or vesicles, without energy consumption^15,16^. Considering their chemical nature, root exudates consist either of primary metabolites including organic acids (e.g*.* citrate, malate, oxalate, lactate), sugars (e.g*.* glucose, fructose) and amino acids (e.g*.* glycine, glutamate, alanine) or secondary metabolites such as phenols, vitamins, glucosinolates, plant hormones, but also enzymes (e.g*.* phytases, phosphatases) and polysaccharides (e.g*.* pectic acid)^15–17^. The amount of carbon (C) released into the rhizosphere greatly depends on the plant species, the genotype, the age, the chemical-physical-biological soil characteristics and biotic or abiotic stresses^18–20^. To cope with P deficiency, the major metabolites that plants release from their roots are organic acids (e.g*.* citrate, malate, oxalate)^21–23^, carbohydrates^24^, phenols (flavonoids, flavonols etc.)^25^ and signal molecules to recruit mycorrhizal symbionts^26^. Additionally, P deficiency triggers an enhanced acidification of the rhizosphere through the active release of protons, mediated by plasma membrane H^+^-ATPases at the expense of ATP^27^. Apart from the energy cost, C and N rich labile compounds released as exudates represent an undoubted source of nourishment for the microorganisms in the rhizosphere. Furthermore, these exudates act also as attractants for microbial pathogens and other microorganisms that could compete for nutrients^16^.

The exudation process is a valid attempt to improve the nutrient acquisition by roots. On the other hand, root exudates represent for the plant also a net energy cost not only for their synthesis but also for their exudation processes. Moreover, if the exudation extent does not correspond to a proportional nutrient mobilization, it is very likely that it may also represent a net loss of energy for the plant, especially in nutrient deficient conditions when exudation is particularly increased. Therefore, a further use of these compounds by the plant could represent an element of a more complex energy-saving strategy adopted by the plants. In fact, it has been demonstrated that some plants have the ability to recapture exudates from the apoplast or directly from the soil solution^16,28^. This phenomenon has been described for amino acids, sugars, polyamines and phytosiderophores, being these latter key players in iron (Fe) uptake in grasses species^16,28,29^. However, to date no root uptake has been documented for organic acids. It has been hypothesized that the transmembrane electrochemical gradient prevents their transport towards the cytoplasm^28^. Considering the mechanism, the root uptake of amino acids, sugars, polyamines and phytosiderophores has been described as an active transport catalyzed by specific transporters and based on the transmembrane cotransport^28,30^. It has been postulated that the root uptake of exudates could be a strategy for the plant to use C, sulfur (S) or N from organic matter^31^. However, this idea is still under debate according to some authors^16^. For most types of exudates released by the plants into the rhizosphere their root uptake remains still unknown^17^. This is especially true in nutrient deficient conditions. Therefore, this study aimed at investigating the effect of P deficiency on the root uptake of five organic compounds often released in the rhizosphere using tomato plants as a model species. To this purpose, stable isotope ratios of C has been used. Stable isotopes are important tools for studying metabolic fluxes and pathways within tissues or cells. By feeding the roots with single labeled exudates at a time, it was possible to measure their uptake by obtaining the δ^13^C value of the root tissue, as well as to ascertain the translocation of the isotope to the shoot. Furthermore, this study revealed the effect of P deficiency on the C isotope fractionation of tomato shoots and roots over time.

## Results

### Plant morphology

**Figure 1:**
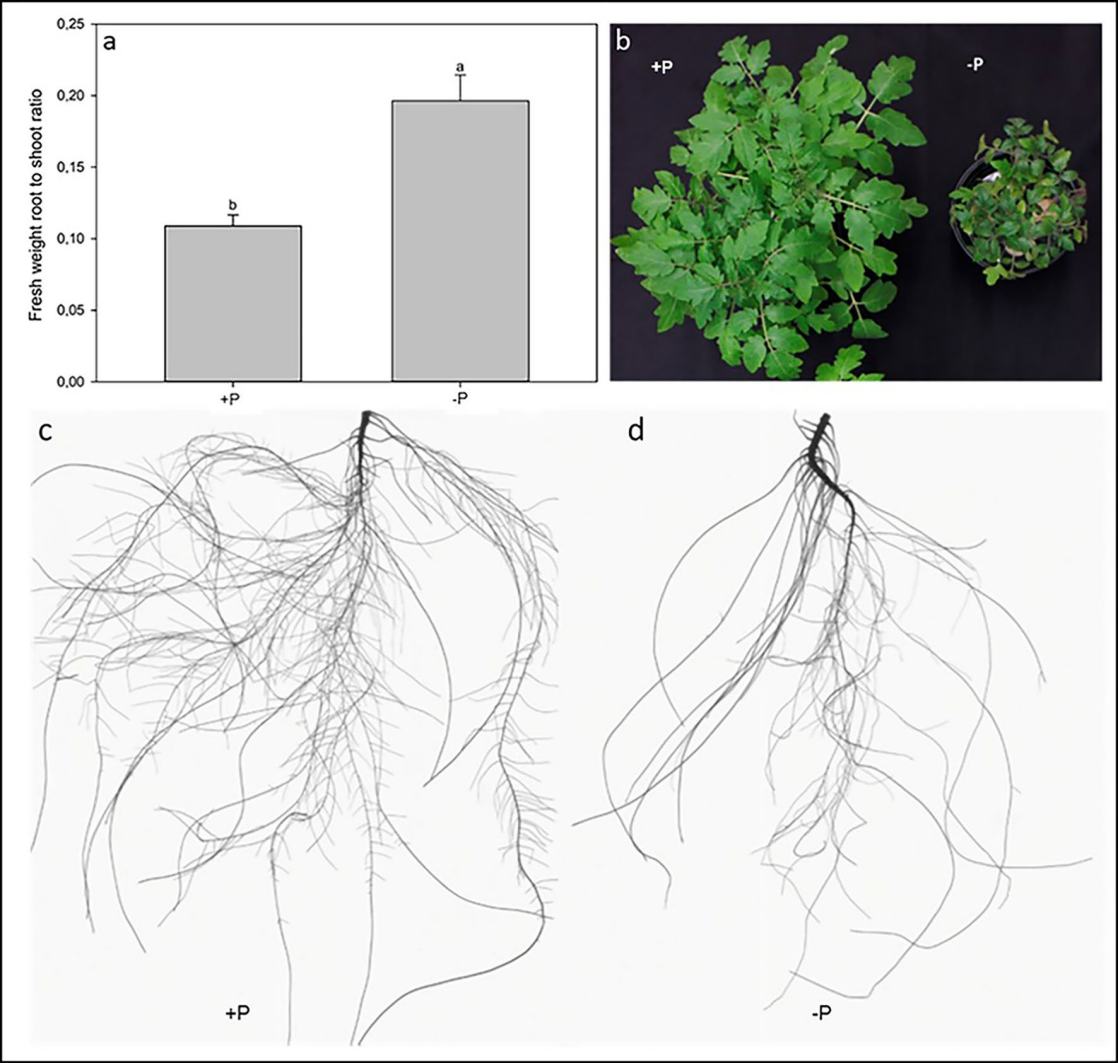
Effects of P deficiency on 31-day-old tomato plants’ morphology. (**a**) Root-to-shoot ratio of tomato plants grown in either control (+ P) or P starvation (− P) conditions; data are presented as mean ± SE, n = 10; lower case letters above the vertical bars indicate statistical significance according to t-test with p < 0.001. (**b**) Shoot biomass of tomato plants grown in + P and − P conditions. (**c**) Representative root system of + P tomato plants. (**d**) Representative root system of − P tomato plants.

Figure 1a shows the root-to-shoot ratio of tomato plants grown in control (+ P) and P deficient (− P) conditions. As expected, − P tomato plants showed a significantly higher root-to-shoot ratio compared to + P plants (+ 55.5%). Indeed, P deficient tomato plants exhibited a clear impaired shoot growth (Fig. 1b) compared to the control plants and a visible reduction of root growth in terms of root elongation, particularly of the lateral roots and root tips development (Fig. 1c,d). However, the reduction in shoot biomass exceeded the reduction of root growth and let to an increased root to shoot ratio of − P plants. Furthermore, P deficient tomato plants revealed an increased root diameter compared to the + P plants (data not shown). − P plants exhibited slightly bluish to reddish colored leaves, i.e*.* the typical symptoms of P deficient plants.

### Carbon fractionation in tomato plants

**Figure 2:**
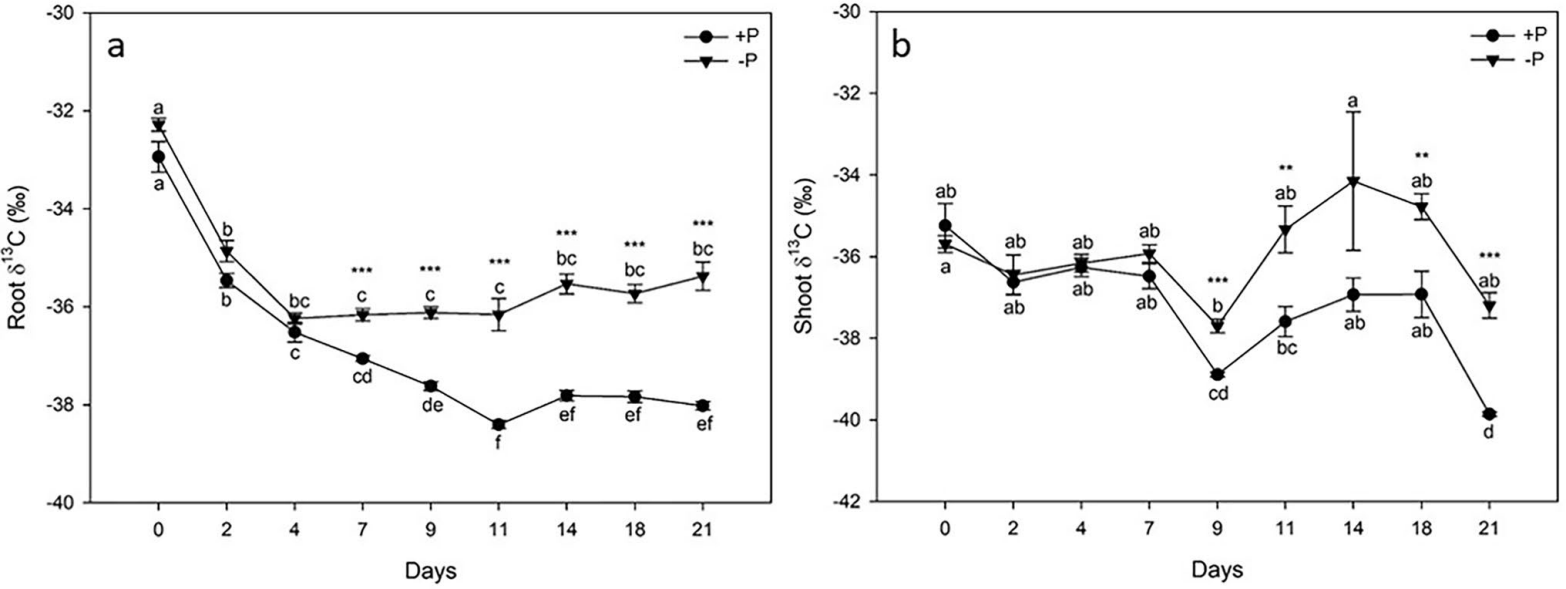
^13^δC values of tomato plants grown in either control (+ P) or P starvation (− P) conditions over time. Day 0 on the x-axis indicates the day in which part of tomato plants were transferred to − P nutrient solutions; control plants were grown in a full nutrient solution (+ P) for the same experimental period. (**a**) ^13^δC values of tomato roots in function of time; data points represent the mean ± SE, n = 5. The statistical significance has been assessed by one-way ANOVA test with Holm–Sidak post hoc test (+ P condition: p value < 0.0001; F value = 132.02; DF = 8; − P condition: p value < 0.0001; F value = 38.51; DF = 8). Mean data of each sampling date, i.e. + P versus − P, have been compared by t-tests (*, p < 0.05; **, p < 0.01, ***, p < 0.001). (**b**) ^13^δC values of tomato shoots in function of time; data points represent the mean ± SE, n = 5. The statistical significance has been assessed by one-way ANOVA test with Holm-Sidak post hoc test (+ P condition: p value < 0.0001; F value = 17.10; DF = 8; − P condition: p value = 0.0043; F value = 3.46; DF = 8). Mean data of each sampling date, i.e. + P versus − P, have been compared by t-tests (*, p < 0.05; **, p < 0.01, ***, p < 0.001).

The carbon isotope fractionation has been monitored over time in both + P and P deficient tomato plants (Fig. 2). Interestingly, the δ^13^C value of both + P and − P roots decreased in the first 4 days without statistically significant differences between + P and − P roots (Fig. 2a). At day 7, the δ^13^C value differed significantly in the two conditions: − P roots exhibited a significantly higher δ^13^C value, thus − P tomato roots were enriched with ^13^C, compared to the + P plants (Fig. 2a). We observed the same trend at every sampling day until harvest, i.e. at day 21. While the δ^13^C values of the − P roots remained constant from day 4 to day 21, the δ^13^C values of + P roots decreased further with time (− 1.34‰ from day 7 to 11) remaining then constant until day 21. Considering the whole experimental period, the δ^13^C values of − P tomato roots decreased by − 3.09‰ from day 0 to day 21, whereas the δ^13^C values of + P tomato roots decreased by − 5.08‰ in the same time period (Fig. 2a).

At shoot level, the carbon isotope fractionation was different over time (Fig. 2b). The δ^13^C values of the shoots of + P and − P tomato plants did not change significantly until day 9. At this point, we observed a sharp decrease in the δ^13^C values of + P shoots, resulting in a significantly lower values compared to those of − P shoots. After day 9, the δ^13^C values of + P tomato shoots increased again, while they decreased again from day 18 to 21 (Fig. 2b). While the δ^13^C value of + P tomato shoots decreased significantly by − 4.61‰ from day 0 to day 21, the carbon fractionation of − P tomato shoots did not change significantly (Fig. 2b). However, from day 9 to day 21, the shoots of − P plants showed significantly higher δ^13^C values respect to + P shoots. At day 14, we encountered an unexpected variability since + P and − P plants did not show any significant differences.

### Uptake of ^13^C-glycine

**Figure 3:**
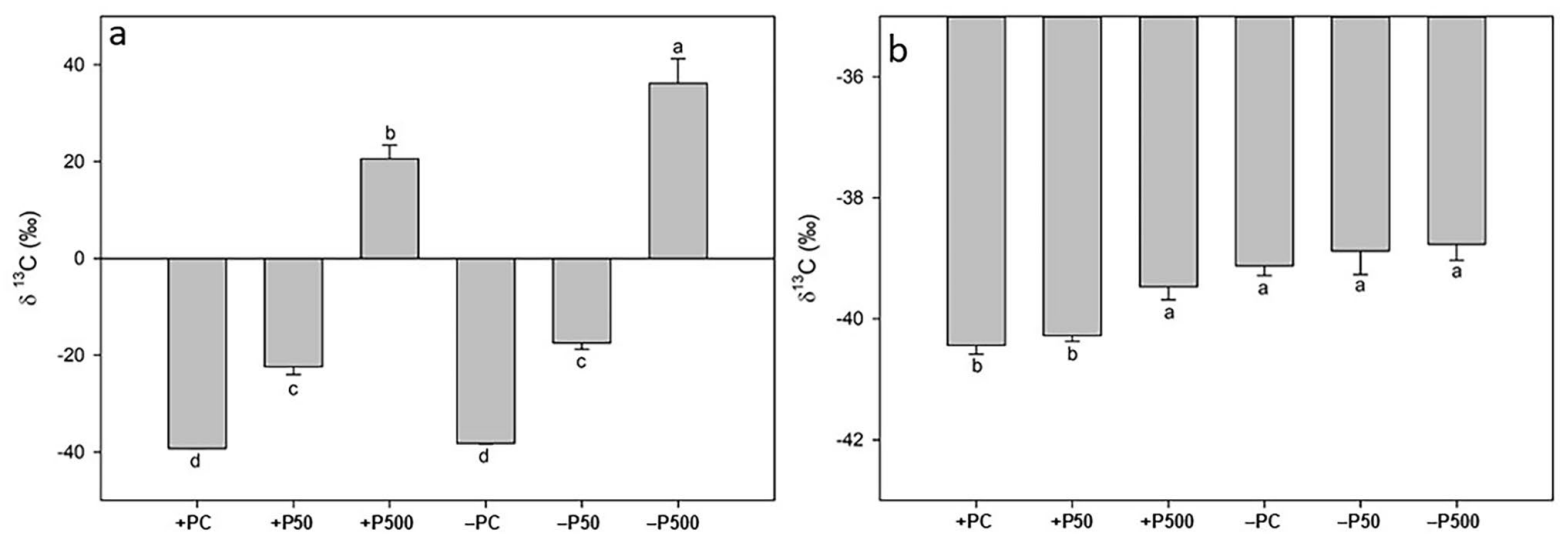
^13^δC values of roots (**a**) and shoots (**b**) of 24-day-old tomato plants grown in a full nutrient solution (+ P) or zero P nutrient solution (− P) after two hours of contact with 0 (+ PC, − PC), 50 (+ P50, P50) and 500 (+ P500, − P500) µmol L− ^1 13^C-labelled glycine uptake solution. Vertical bars represent the mean ± SE, n = 7; lower case letters above the vertical bars indicate statistical significance according to one-way ANOVAs comparing averages through Holm-Sidak post hoc tests (roots (**a**): p value < 0.001; F value = 308.33; DF = 5; shoots (**b**): p value < 0.001; F value = 12.77; DF = 5).

Figure 3 reports the δ^13^C values of 24-days-old (of which 14 days in + P and − P condition) intact tomato plants (expressed per roots and shoots) after 2-h contact of roots with the uptake solution containing ^13^C labelled glycine at different concentrations. Glycine affected the δ^13^C values of both + P and − P plants and particularly the roots in both conditions (Fig. 3a). Indeed, + P and − P plants treated with the highest ^13^C-labelled glycine revealed the significantly highest ^13^C enrichment in their roots (+ 59.8‰ and + 74.4‰ compared to their respective controls, i.e. + PC and − PC). Furthermore, − P roots treated with the highest concentration of ^13^C-labelled glycine exhibited the significantly highest δ^13^C value among all the treatments indicating that P deficiency further triggers glycine uptake by tomato roots (Fig. 3a).

It is interesting to note that glycine was taken up also when applied in lower concentrations (50 µmol L^−1^) without significant differences between + P50 and − P50 in shoots. The lowest δ^13^C value can be observed in + PC and − PC plants, which never came in contact with the ^13^C labelled glycine solution. Glycine supply led to a ^13^C enrichment in the shoots of tomato plants, but to a much lower extent compared to its accumulation in roots (Fig. 3b). It is noteworthy that − PC shoots showed a significant higher δ^13^C value (+ 1.27‰) compared to + PC. Furthermore, shoots of + P plants exhibited a ^13^C enrichment only when treated with the highest concentration of glycine (+ 0.92‰, + P500, Fig. 3b). The same enrichment has been observed for all the − P conditions, i.e. − PC, − P50 and − P500, without statistical significance among them.

### Uptake of ^13^C-glucose

**Figure 4:**
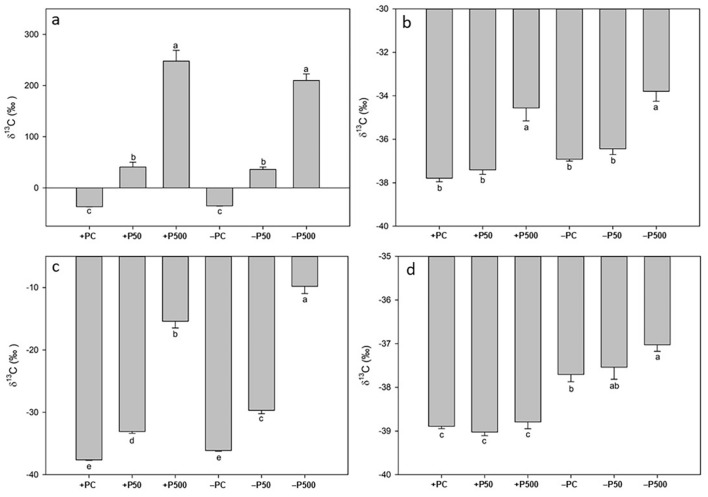
^13^δC values of roots (**a** and **c**) and shoots of 24-dayold tomato plants grown in a full nutrient solution (+ P) or zero P nutrient solution (− P) after two hours of contact with 0 (+ PC, − PC), 50 (+ P50, P50) and 500 (+ P500, − P500) µmol L^−1 13^C-labelled glucose (**a** and **b**) and fructose (**c** and **d**) uptake solutions. Vertical bars represent the mean ± SE, n = 7; lower case letters above the vertical bars indicate statistical significance according to one-way ANOVAs comparing averages through Holm–Sidak post hoc tests (roots glucose (**a**): p value < 0.001; F value = 126.65; DF = 5; shoots glucose (**b**): p value < 0.001; F value = 21.92; DF = 5; roots fructose (**c**): p value < 0.001; F value = 278.02; DF = 5; shoots fructose (**d**): p value < 0.001; F value = 26.75; DF = 5).

Figure 4a,b shows the δ ^13^C values of tomato roots and shoots after the uptake of ^13^C-labelled glucose solutions. We observed a similar trend as the one obtained with glycine (Fig. 3) even though glucose led to a high ^13^C enrichment in roots already at low concentrations (i.e. 50 µmol L^−1^) and in both conditions (+ 77.7‰ and + 284‰ in + P50 and − P50, respectively, compared to their respective controls). Tomato roots treated with the highest glucose concentration, i.e*.* + P500 and − P500, exhibited the significantly highest values of δ^13^C, being fivefold higher than their respective controls, + PC and − PC (Fig. 4a).

Regarding the δ^13^C values determined in tomato shoots, noteworthy significant ^13^C enrichments were observed only in plants supplied with the highest concentration of glucose, i.e. + P500 and − P500 (+ 3.23‰ and + 3.12‰, respectively compared to their respective controls, Fig. 4b). They exhibited the highest δ^13^C over all other treatments (Fig. 4b). No significant variations were recorded among the other treatments.

### Uptake of ^13^C-fructose

**Figure 5:**
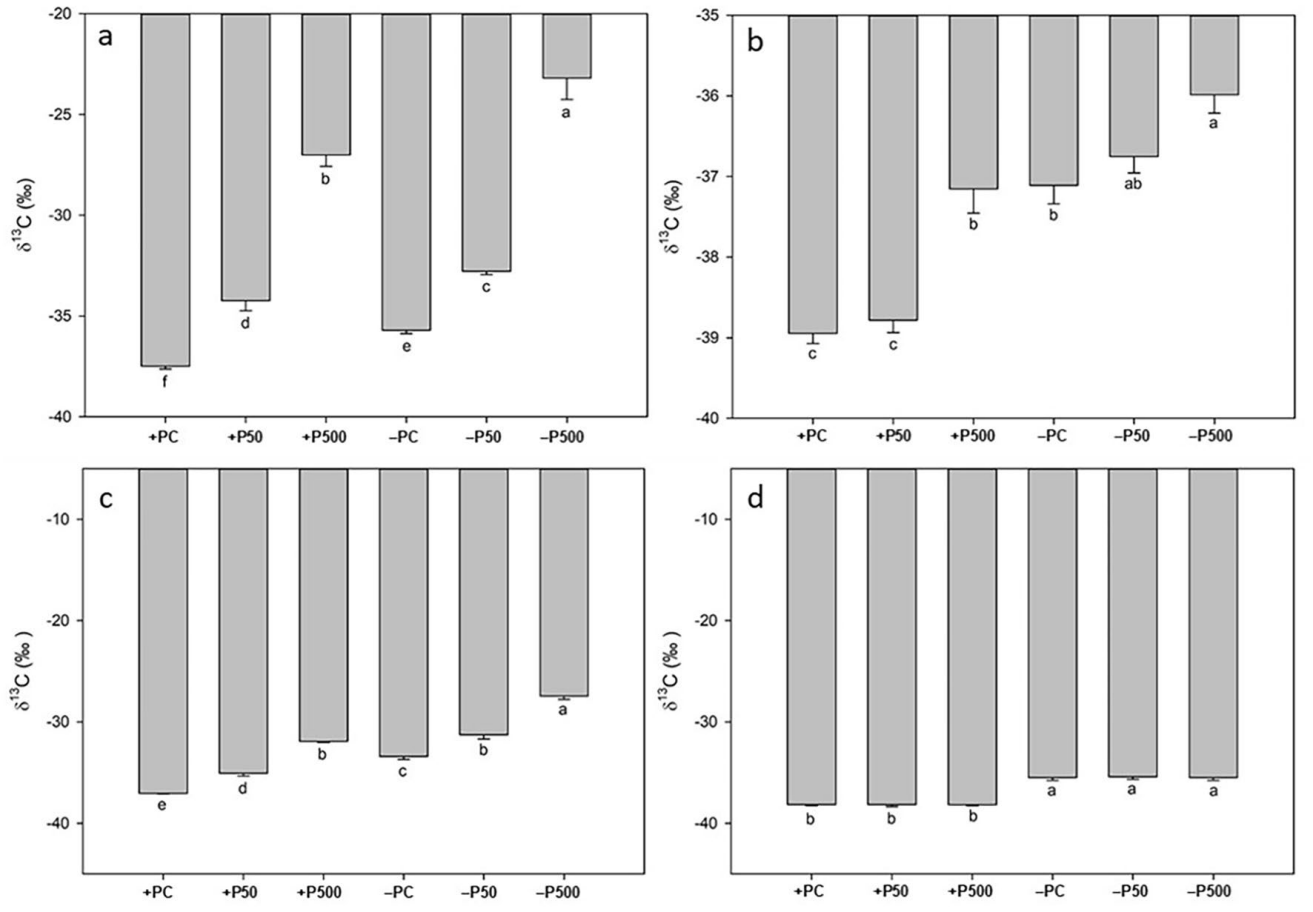
^13^δC values of roots (**a** and **c**) and shoots (**b** and **d**) of 24-day-old tomato plants grown in a full nutrient solution (+ P) or zero P nutrient solution (− P) after two hours of contact with 0 (+ PC, − PC), 50 (+ P50, P50) and 500 (+ P500, − P500) µmol L^−1 13^C-labelled citrate (**a** and **b**) and malate (**c** and **d**) uptake solution. Vertical bars represent the mean ± SE, n = 7; lower case letters above the vertical bars indicate statistical significance according to one-way ANOVAs comparing averages through Holm–Sidak post hoc tests (roots citrate (**a**): p value < 0.001; F value = 161.80; DF = 5; shoots citrate (**b**): p value < 0.001; F value = 29.74; DF = 5; roots malate (**c**): p value < 0.001; F value = 137.36; DF = 5; shoots malate (**d**): p value < 0.001; F value = 47.76; DF = 5).

Figure 4c,d shows the different δ^13^C value of tomato roots and shoots after 2 h of contact with an uptake solution containing ^13^C-labelled fructose. In both + P and − P plants, ^13^C enrichment of the roots increased with increasing fructose concentration supplied in the uptake solution (Fig. 4c). In particular, the significantly highest value of δ^13^C was found in − P tomato roots treated with the highest fructose concentration (− P500, + 26.3‰ compared to its control, − PC, Fig. 4c), followed by roots of + P plants treated with the same sugar concentration (+ P500, + 22.2‰ compared to its control, + PC, Fig. 4c). Considering the treatment with 50 µmol L^−1^ fructose, we observed a significant higher ^13^C enrichment in − P roots than in + P roots (+ 3.38‰ in − P50 compared to + P50, Fig. 5a). Furthermore, both + P50 and − P50 treatments showed a significantly higher δ^13^C values than their respective controls. No significant differences were revealed between the two controls (+ PC and − PC).

Tomato shoots were also enriched in ^13^C (Fig. 4d). It is interesting to note that all − P shoots, display significantly higher δ^13^C values compared to + P shoots (on average + 1.48‰, Fig. 4d). The treatment with 500 µmol L^−1^ fructose led to a slightly higher, yet statistically significant, ^13^C enrichment in the − P shoots when compared to their control (− P500 + 0.68‰ respect to − PC, Fig. 4d). On the other hand, − P plants treated with 50 µmol L^−1^ displayed no significant δ^13^C value alterations when compared to their control. No noteworthy changes occurred between the + P tomato shoots.

### Uptake of ^13^C-citrate

Figure 5a,b reports the different δ^13^C values from tomato roots and shoots after contact via the root system with ^13^C-labelled citrate solutions at different concentrations. Citrate affected the ^13^C fractionation of both + P and − P roots significantly among all treatments (Fig. 5a). Indeed δ^13^C values increased significantly with increasing citrate concentration supplied in the uptake solution, showing the same trend in both conditions (Fig. 5a). Control treatment of − P and + P plants, i.e*.* + PC and − PC, revealed that − P roots displayed significantly higher δ^13^C values (+ 1.79‰ of − PC compared to + PC, Fig. 5a). Supplying the roots with 500 µmol L^−1 13^C-labelled citrate solutions led to a significantly higher uptake of citrate in the − P500 plants compared to both their − P control and their equal treated + P counterpart (+ 12.5‰ of − P500 compared to − PC and + 3.81‰ compared to + P500, Fig. 5a). Roots of tomatoes treated with 50 µmol L^−1^ solutions showed overall lower δ^13^C values than the 500 µmol L^−1^ treated roots, however the trend was the same in both conditions: − P50 roots exhibited a significant ^13^C enrichment of 2.92‰ and 1.45‰ when compared to − PC and + P50, respectively (Fig. 5a).

Figure 5b shows the δ^13^C values of tomato shoots after exposure of roots to the ^13^C-labelled citrate uptake solutions. Our results showed that − PC shoots exhibited significantly higher δ^13^C values (+ 1.84‰) respect to the + PC shoots. Furthermore, the treatment with 500 µmol L^−1^ labelled citrate affected significantly the δ^13^C values of both + P and − P shoots, although the shoots of − P showed a higher ^13^C enrichment (+ 1.17‰) when compared to the + P500 shoots (Fig. 5b). The exposure to 50 µmol L^−1^ solutions resulted in a significant higher (+ 2.03‰) ^13^C accumulation in − P50 shoots compared to the respective control (+ P50).

### Uptake of ^13^C-malate

δ^13^C values of roots and shoots after exposure to different concentrations of ^13^C-labelled malate solutions are displayed in Fig. 5c,d. Malate affected the ^13^C fractionation of both + P and − P tomato roots. Again, as observed for labelled citrate, malate led to an increasing ^13^C enrichment with increasing concentration of malate supplied, both in roots and in shoots. When comparing the control treatments of + P and − P, we observed a significantly higher δ^13^C value in the − P root (+ 3.64‰). Immersing the roots in 500 µmol L^−1 13^C-labelled malate solutions resulted in the highest ^13^C enrichment in − P plants (− P500 + 5.95‰ and + 4.47‰ compared to − PC and + P500, respectively). Interestingly the − P50 treatment showed the same enrichment as the + P500 plants (Fig. 5c). However, when comparing the δ^13^C values of − P50 to its control (− PC) and to its equally treated control plant (+ P50), the − P50 roots revealed significantly higher values (+ 2.13‰ vs. − PC and + 3.79‰ vs. + P50, Fig. 5c).

Figure 5d displays the δ^13^C values determined in tomato shoots after root exposure to the different ^13^C-labelled malate solutions. Interestingly, the malate treatments showed no effect on the ^13^C fractionation. The only significant difference obtained was that all − P shoots showed significantly higher δ^13^C values compared to the + P shoots (average + 2.69‰), as observed earlier in the carbon fractionation experiment (Fig. 2). No significant differences occurred within the + P and − P treatments.

### Carbon uptake derived from ^13^C metabolites

Table 1 shows the µg C derived from the^13^C exudate source of tomato roots and shoots after exposure with the ^13^C-labelled exudate solutions. The overall trend is the same as the one described for the δ^13^C values (Figs. 3, 4 and 5). However, in the case of roots treated with glycine, we could detect a significant higher amount of source derived C even in the treatment with the lower concentration (+ 37% − P50 vs + P50, Table 1). On the other hand, in the shoots the + P500 displayed a significant higher amount of source derived C when compared to − P500. The treatment with ^13^C labeled glucose lead to exact the same trend in both roots and shoots as the one described for the δ^13^C values (Fig. 4a,b). Fructose ^13^C treatment leads to the same trend in roots as described for δ^13^C values (Fig. 4c, Table 1). However, in shoots, no significant alteration was found, although the trend of the averages seems the same as for the δ^13^C values (Fig. 4d, Table 1). At the root level citrate and malate uptake resulted significant higher (+ 37% for both) only at the higher concentration (500 µmol L^−1^) when comparing − P500 and + P500 (Table 1). However, in the shoots treated with citrate the trend was the same as described with the δ^13^C values (Fig. 5b, Table 1). On the other hand, the shoots of the − P50 malate treatment revealed a 569% higher amount of C derived from the source when compared to the + P50 treatment (Table 1).

## Discussion

When the available P fractions are not adequate in soil, several typical symptoms appear at the plant level including an increased root to shoot ratio^32,33^, an alteration of the root morphology^32^ and an accumulation of anthocyanins and other pigments^34–36^ with a consequent bluish color of the leaves. The − P tomato plants used in the present work showed all these symptoms (Fig. 1a–d) indicating the onset of the typical responses to the nutritional disorder. It is worthy to note with respect to the root architecture that the root development is a very complex process, often genotype-dependent, involves several hormone signaling pathways and often leads to an enhanced root exudation^16,37,38^. This latter is a generally well-known phenomenon in nutrient disorders, especially in P deficiency^15,16^. However, even if up to approx. 30% of the photosynthetically assimilated C is translocated to roots and released into the rhizosphere, exudates get easily and readily (i) metabolized by microorganisms with half-lives often shorter than one hour^39^ or (ii) bound to the solid soil mineral and organic colloids^15,17^. Thus, root exudation could represent a great energy loss exacerbating the trade-offs between investments and returns in terms of nutrients. The exudates’ reacquisition by roots is potentially possible, even though only limited information is available^16,17,28,30,40^. In this work it has been postulated that plants can recapture some of the released C also triggering some specific exudate uptake mechanisms, as for instance in nutrient deficient conditions^16^.

Up to date no root reacquisition of exuded carboxylates has been demonstrated^29,41^, even if they are released in abundant quantities in P deficiency. Most of the previous studies have suggested, when detectable^34^, the functionality of only a root uptake mechanism of mono-valent organic acids (e.g. acetic acid). A very recent study however identified a bidirectional aluminum-malate transporter, which may be also involved in root malate uptake^42^. Results here presented, reveal that tomato plants are able to take up citrate from the uptake solution (Fig. 5, Table 1). Moreover, the process is affected by the nutritional status of the plant, being significantly enhanced in − P plants. Since citrate, as a root exudate, plays a crucial role in P mobilization processes, we suggest that it might be of great interest for the plant to recapture the lost C from citrate itself, also to minimize the energy loss. Higher δ^13^C values and amounts of exudate derived C were detected also in shoots (Fig. 5, Table 1). Therefore, we imply that the ^13^C from citrate has been translocated to the shoots, either incorporated in citrate or in other metabolized forms. Considering the key role played by some organic molecules (including citrate) with a high affinity for metals in the xylem translocation of these elements to the shoot^3^, this result appears to be even more of particular interest. Regarding malate, root uptake has been detected, which was similarly to citrate enhanced in − P tomato plants (Fig. 5a, Table 1). Increased translocation of the ^13^C to the shoots has been observed in − P plants supplied with ^13^C-malate just in the lower concentration treatment (Table 1). This might indicate that the products of malate’s metabolization remain in the roots in some cases, at least for the 2 h of the experiment.

In the present work the uptake of the proteinaceous amino acid glycine has also been analyzed. Amino acids are among the most exuded metabolites^24^ and particularly involved in N deficient conditions. In fact, amino acids can even act as N source for the plant if no other sources are available^43^. Although it seems that proteinaceous amino acids have generally no significant role in nutrient mobilisation^44^, there is some recent evidence indicating the glycine involvement in the iron mobilization ^45^. In our study the amino acid glycine was taken up by the tomato plants (Fig. 3a,b, Table 1). This phenomenon was significantly enhanced in the + P500 and − P500 treatment when considering the δ^13^C values. The reacquisition of amino acids^46^ and other N-containing compounds^29^ has been already shown in literature. The ^13^C from glycine was also translocated to the shoots, but only when the amino acid was supplied at the final concentration of 50 µmol L^−1^ (Fig. 3b) In terms of µg C derived from the glycine source both − P treatments showed a significant higher uptake than the + P (Table 1). In the last years, molecular approaches identified more than 10 amino acid transporter families, that are able to take up amino acids also from the rhizosphere^47^. However, results here reported show for the first time that P deficiency can boost the uptake of these compounds, at least in tomato plants. Since plants lose lots of C, N and energy by exudation, it is suggested that the re-acquisition here presented could represent a mechanism for the plant to regain some of the energy and C. Recently it has been shown that also wheat plants are able to take up exuded organic N-containing compounds^29^.

In our study, results show that glucose is taken up by the tomato roots. However, this phenomenon is not affected by the plant nutritional status (Fig. 4a, Table 1). Only the higher concentration of glucose led to a higher δ^13^C value. Glucose is not known to play a role in increasing nutrient uptake efficiency but once released in the external environment, it represents a fundamental C source for beneficial bacteria such as Plant Growth Promoting Rhizobacteria (PGPR) and mycorrhizal fungi^48^. The lack in increase of glucose uptake in P deficiency could be explained by the positive effect that glucose has on microorganism population and therefore the plant favors exudation over reacquisition. Microorganisms might return the favor by mobilizing P for the plant as widely described in P deficient conditions^3^. Translocation to the shoots of glucose was observed only when supplied at the highest concentration (500 µmol L^−1^), regardless of the nutritional status of the plants (+ P or − P) (Fig. 4b, Table 1). Differently, the results here presented show that that fructose uptake by roots was affected by P deficiency. The tomatoes grown in − P and exposed to ^13^C-labelled fructose showed always a significantly higher δ^13^C value when compared to their respective controls (Fig. 4c, Table 1). In this respect, it is interesting to note that a monosaccharide transporter superfamily has been already identified in plants therefore supporting the hypothesis of a root uptake process of monosaccharides^49^. This idea is further supported by the findings obtained by using ^13^C isotopologues in wheat^28^. Furthermore, this possibility has been also confirmed in two other older studies conducted using maize as a model plant^30,40^. The differences in uptake between the two sugars needs further molecular investigation to be fully elucidated. It is interesting to note that all the evidence concerning the possibility of a root uptake of sugars have been achieved using plants fully supplied with nutrients. Therefore, the effect on the modulation of the process related to the plant nutritional state (e.g. nutrient deficiency) here highlighted is definitely new.

Interestingly and unexpectedly, − P plants showed a different ^13^C fractionation compared to + P plants. Therefore, we conducted a time course experiment in which we assessed the δ^13^C values of shoots and roots separately over time in + P and − P conditions (Fig. 2a,b). The δ^13^C values of roots decreased significantly in both conditions during the first 4 days. Afterwards, the δ^13^C values of + P plants continued to decrease, while the δ^13^C values of − P plants remained constant. This trend resulted in significantly higher δ^13^C values of − P roots compared to + P roots at the end of the experiment. Essentially the same phenomenon has been observed at the shoot level between + P and − P plants. However, shoots of − P plants presented a significantly higher δ^13^C values only after 9 days and not after 7 days as it is shown in the roots. Carbon fractionation occurs during photosynthetic CO_2_ assimilation where ribulose-1,5-bisphosphate carboxylase/oxygenase (Rubisco) is the first enzyme of the metabolic pathway. This enzyme catalyzes the CO_2_ assimilation using preferably ^12^C over ^13^C as substrate. In addition, ^12/13^CO_2_ fractionation occurs during diffusion into the cell^50,51^. Phosphorus starvation in plants often leads to a disruption of hydraulic conductance due to stomata closure^52–54^. Indeed, when the stomata are closed, plants use the CO_2_ which is already present in the leave cells and therefore it is very likely that Rubisco can consume also more ^13^C as substrate. We hypothesize that the δ^13^C values of − P plants are therefore significantly higher than + P plants once the plant, sensing the shortage, starts the stress response strategy. To date there are only a few studies explaining that nutrient deficiency can impact the C fractionation^51^. An experiment in which climber plant species were grown with limited P supply revealed that *Pharbitis nil* L. could change its C source, which came also from the CO_2_ supplied by the transformation from HCO_3_ through carbonic anhydrase^55^. Another study revealed that in maize the δ^13^C values increased with decreasing N availability^56^. Furthermore, research conducted with microalgae revealed that P and N deficiency can result into more positive δ^13^C values^57^.

## Conclusions

The present study examined the effect of P deficiency on the root uptake of specific organic molecules belonging to root exudates’ classes. Additionally, we unraveled that P deficiency led to ^13^C enrichment over time both in tomato shoots and roots by + 2.66‰ and + 2.64‰ respectively, when compared to + P plants. Our results imply that tomato plants were able to reacquire fundamental metabolites and therefore exhibited direct control over the amount of C present in the rhizosphere. Interestingly, − P tomato plants showed an increased capacity to take up specific exudates in some cases (i.e*.* + 37% citrate and malate when comparing − P500 vs + P500 roots). This indicates that the plants maximized the C trade off and recaptured the lost C to a greater extent when subjected to unfavorable conditions such as P starvation. This could be a part of a more complex energy/C saving strategy of plants, especially pronounced in nutrient deficiencies. However, being well known that the root exudation is species/genotype dependent and affected by several environmental factors, further investigations are necessary to characterize the effect of these aspects/conditions. Furthermore, the molecular mechanisms underlying the uptake of these exudates need still to be elucidated.

### Materials and methods

#### Growing condition

Tomato (*Solanum lycopersicum* L., cultivar Marmande) seeds were germinated in plastic boxes containing several layers of tissue paper moistened with 0.05 mmol L^−1^ CaSO_4_, while maintaining darkness and 25 °C for 9 days. Uniform seedlings were selected and transferred into plastic pots (10 plants per pot) containing 1.5 L of aerated nutrient solution with the following composition (mmol L^−1^): 2 Ca(NO_3_)_2_, 0.7 K_2_SO_4_, 0.1 KH_2_PO_4_, 0.1 KCl, 0.5 MgSO_4_; and (µmol L^−1^): 10 H_3_BO_3_, 0.5 MnSO_4_, 0.2 CuSO_4_, 0.5 ZnSO_4_, 0.01 (NH_4_)_6_Mo_7_O_24_, and 5 Fe(III)-EDTA. The nutrient solution was renewed every 3 days. Plants were grown in a climatic growth chamber with day/night cycle of 14/10 h, temperature regime of 24/19 °C, light intensity of 250 µmol m^−2^ s^−1^ at plant level and a relative humidity of 70%. After 10 days of growth in the full nutrient solution, half of the tomato plants were transferred in a phosphorus zero (− P) nutrient solution. The other half was kept in a full nutrient solution as control plants (+ P).

### Long term experiment

Ten-day-old tomato plants were grown for another 21 days either in a full nutrient solution (+ P) or in a zero P (− P) solution. Plants were harvested 0, 2, 4, 7, 9, 11, 14, 18 and 21 days: shoots were detached from the roots and both were dried at 70 °C for 3 days until they reached constant weight. The dry biomass was ground with a Mix Miller MM 400 (Retsch, Italy) at an oscillation frequency of 30 s^−1^ for 3 min per sample. The completely ground and homogeneous shoots and roots were used for the δ^13^C analysis. Experiments were run with 5 replicates.

### Re-uptake of Exudates

Ten-day-old tomato plants were grown for another 14 days either in a full nutrient solution (+ P) or in a zero P (− P) solution. After 14 days, single tomato plants were immersed in 20 mL solutions containing ^13^C labelled root exudates: glycine (glycine-1-^13^C, 99 atom % ^13^C Sigma Aldrich, Italy), glucose (D-glucose-^13^C_6_, ≥ 99 atom% ^13^C, Sigma Aldrich, Italy), fructose (D-fructose-1-^13^C, 99 atom % ^13^C, Sigma Aldrich, Italy), citrate (citric acid-1,5-^13^C_2_, atom 98% ^13^C, Sigma Aldrich), and malate (DL-malic acid-2-^13^C, 99 atom % ^13^C, Sigma Aldrich, Italy). δ^13^C was measured in both roots and shoots after 2 h. In brief, − P and + P tomato plants were carefully removed from the pots and their root systems was immersed into an aerated 0.5 mmol L^−1^ CaSO_4_ solution for 15 min. Afterwards, their roots were carefully dried with tissue paper. Each plant was transferred to a small pot immersing the complete root system in 20 mL aerated solution containing either 0, 50 or 500 µmol L^−1^ of the above-mentioned root exudates (Table 2). Each solution additionally contained 1 mg L^−1^ Micropur (Katadyn, Italy) to prevent microbial degradation of the target molecules. The pots were covered with aluminum foil to create dark conditions for roots.

**Table 1:** Carbon deriving from the ^13^C exudate source of roots and shoots of tomatoes grown in either + P or − P conditions. Data represent mean (µg) ± SE, n = 7; lower case letters indicate statistical significance of different treatments of the same molecule (+ P50 vs + P500 vs − P50 vs − P500). Statistical analysis performed: one-way ANOVAs comparing averages through Holm-Sidak post hoc tests. The last column displays the p-values, F-values and DF-values of the ANOVAs.

Root (µg)	+ P50	+ P500	− P50	− P500	p, F, DF
Glycine	38.7 ± 3.63d	137 ± 6.40b	54.7 ± 3.59c	196 ± 13.3a	< 0.001, 22.2, 3
Glucose	30.3 ± 3.60b	111 ± 8.26a	32.0 ± 2.15b	110 ± 5.72a	< 0.001, 20.3, 3
Fructose	43.3 ± 2.64d	211 ± 10.3b	70.3 ± 5.69c	287 ± 12.7a	< 0.001, 175, 3
Citrate	12.9 ± 1.98c	41.6 ± 2.26b	13.3 ± 0.74c	57.0 ± 4.87a	< 0.001, 21.4, 3
Malate	30.8 ± 0.69b	21.9 ± 1.90c	26.2 ± 3.02bc	52.3 ± 2.19a	< 0.001, 41.1, 3

Shoot (µg)	+ P50	+ P500	− P50	− P500	p, F, DF
Glycine	2.40 ± 0.49c	21.6 ± 3.27a	11.8 ± 5.27b	10.54 ± 3.7b	0.009, 5.66, 3
Glucose	2.12 ± 0.72b	11.0 ± 2.04a	1.79 ± 0.27b	6.48 ± 0.94ab	< 0.001, 10.1, 3
Fructose	12.3 ± 4.73	32.9 ± 9.59	27.0 ± 18.1	34.4 ± 7.60	ns
Citrate	6.96 ± 0.66d	23.5 ± 4.81bc	15.6 ± 3.45c	61.8 ± 10.3a	0.015, 10.3, 3
Malate	13.6 ± 4.04c	122 ± 11.8a	87.1 ± 7.61b	96.8 ± 6.28a	< 0.001, 19.3,3

**Table 2:** Experimental setup to determine the uptake of different ^13^C-labelled exudates.

Sample ID	Description
+ PC	+ P plants immersed in water + 1 mg L^−1^ micropur
+ P50	+ P plants immersed in 50 µmol L^−1^ of the target uptake compounds1 mg L^−1^ micropur
+ P500	+ P plants immersed in 500 µmol L^−1^ of the target uptake compounds1 mg L^−1^ micropur
− PC	− P plants immersed in water + 1 mg L^−1^ micropur
− P50	− P plants immersed in 50 µmol L^−1^ of the target uptake compounds 1 mg L^−1^ micropur
− P500	− P plants immersed in 500 µmol L^−1^ of the target uptake compounds 1 mg L^−1^ micropur

After 2 h, the plants were removed from the uptake solution and washed five times in deionized water to remove any possible isotope labelled molecule residue from the roots surface. Roots were separated from the shoots and both were weighed. The plant tissue was dried at 70 °C for 3 days until constant weight was reached. The dry plant material was ground with a Mix Miller MM 400 (Retsch, Italy) at an oscillation frequency of 30 s^−1^ for 3 min per sample. The completely ground shoots and roots were used for the δ^13^C analysis. Experiments were run with 7 replicates.

### Isotope analysis

δ^13^C analysis was performed using an Isotope Mass Spectrometer (Delta V Thermo Scientific, Germany) following total combustion in an Elemental Analyzer (EA Flash 1112 Thermo Scientific, Germany). Approximately 0.25 mg of subsamples were weighed into tin capsules and analysed for C concentration and δ^13^C. The tin capsules were placed into the Elemental Analyser with an oxidation furnace temperature of 1020 °C and a reduction furnace temperature of 900 °C; a Mg(ClO_4_)_2_ trap removed the produced H_2_O. The isotope ratios were expressed in δ‰ versus V-PDB (Vienna–Pee Dee Belemnite) for of δ^13^C according to the following Eq. (1):

1 $$\updelta ^{{{13}}} {\text{C}}\textperthousand \, = \, \left[ {\left( {{\text{R}}_{{{\text{sample}}}} {-}{\text{ R}}_{{{\text{standard}}}} } \right)/{\text{R}}_{{{\text{standard}}}} } \right] $$

where R expresses the ratio between the heavier *vs*. the lighter isotope, R_sample_ is the isotope ratio measured for the sample and R_standard_ is the isotope ratio of the international standard. The isotope values were calculated against international reference materials: Caffeine IAEA-600 (δ^13^C = − 27.77‰; IAEA-International Atomic Energy Agency, Vienna, Austria). For the quality control of the analysis, 8 samples of caffeine IAEA-600 and 8 samples Urea (δ^13^C = -40.81‰; IVA Analysentechnik, Messbach, Germany) were analysed at regular intervals with the samples. The reproducibility of the C isotopic determination, based on 10 replicate analyses of the working standard, was ± 0.2‰.

The amount in µg of ^13^C derived from the metabolite source solution (ƒ_r_) was obtained by an isotopic mass balance method as follows^58^:

2 $$ f_{{\text{r}}} \left( {\upmu {\text{g}}} \right) = \left( {\left( {\updelta ^{{{13}}} {\text{C}}_{{{\text{TP}}}} -\updelta ^{{{13}}} {\text{C}}_{{\text{C}}} } \right)/\left( {\updelta ^{{{13}}} {\text{C}}_{{\text{S}}} -\updelta ^{{{13}}} {\text{C}}_{{\text{C}}} } \right)} \right)*{\text{g C}}*{1}000000 $$

where δ^13^C_TP_ corresponds to the δ^13^C value of the shoot or root of the plants treated with the ^13^C labeled exudate solutions, δ^13^C_C_ corresponds to the δ^13^C value of plants treated with deionized water and 1 mg L^−1^ Micropur, δ^13^C_S_ corresponds to the δ^13^C value of the metabolite labeled with ^13^C and g C corresponds to the gram of C present in the plant tissues.

### Statistical analysis

The results are presented as means ± standard error (SE). Statistical analysis was performed using SigmaPlot 12 on Windows 10 64 bit. Two conditions were compared through t-tests. p-values of < 0.05 were treated as statistically significant differences. Comparisons between 3 and more conditions were analyzed by one-way analysis of variance (ANOVA), and means were compared using Holm-Sidak post hoc test at p < 0.05 to determine the significance of differences found.
